# Unsupervised Deep Anomaly Detection in Chest Radiographs

**DOI:** 10.1007/s10278-020-00413-2

**Published:** 2021-02-08

**Authors:** Takahiro Nakao, Shouhei Hanaoka, Yukihiro Nomura, Masaki Murata, Tomomi Takenaga, Soichiro Miki, Takeyuki Watadani, Takeharu Yoshikawa, Naoto Hayashi, Osamu Abe

**Affiliations:** 1 Department of Computational Diagnostic Radiology and Preventive Medicine, The University of Tokyo Hospital, 7-3-1 Hongo, Bunkyo-ku, Tokyo, Japan; 2 Department of Radiology, The University of Tokyo Hospital, 7-3-1 Hongo, Bunkyo-ku, Tokyo, Japan; 3 Department of Management, Japan University of Economics, 3-11-25 Gojo, Dazaifu-shi, Fukuoka, Japan; 4 Division of Radiology and Biomedical Engineering, Graduate School of Medicine, The University of Tokyo, 7-3-1 Hongo, Bunkyo-ku, Tokyo, Japan

**Keywords:** Chest radiograph, Variational autoencoder, Generative adversarial network, Deep learning, Unsupervised learning, Anomaly detection

## Abstract

The purposes of this study are to propose an unsupervised anomaly detection method based on a deep neural network (DNN) model, which requires only normal images for training, and to evaluate its performance with a large chest radiograph dataset. We used the auto-encoding generative adversarial network (α-GAN) framework, which is a combination of a GAN and a variational autoencoder, as a DNN model. A total of 29,684 frontal chest radiographs from the Radiological Society of North America Pneumonia Detection Challenge dataset were used for this study (16,880 male and 12,804 female patients; average age, 47.0 years). All these images were labeled as “Normal,” “No Opacity/Not Normal,” or “Opacity” by board-certified radiologists. About 70% (6,853/9,790) of the Normal images were randomly sampled as the training dataset, and the rest were randomly split into the validation and test datasets in a ratio of 1:2 (7,610 and 15,221). Our anomaly detection system could correctly visualize various lesions including a lung mass, cardiomegaly, pleural effusion, bilateral hilar lymphadenopathy, and even dextrocardia. Our system detected the abnormal images with an area under the receiver operating characteristic curve (AUROC) of 0.752. The AUROCs for the abnormal labels Opacity and No Opacity/Not Normal were 0.838 and 0.704, respectively. Our DNN-based unsupervised anomaly detection method could successfully detect various diseases or anomalies in chest radiographs by training with only the normal images.

## Introduction

In recent years, deep neural network (DNN)–based approaches have made remarkable advances in the field of computer-aided diagnosis/detection (CAD) for chest radiographs [1–8]. Most of these works have been carried out in supervised learning, which is a type of training based on labels corresponding to the inputs, such as the type of disease and the location of each lesion. However, such CAD systems based on supervised learning techniques (simply referred to as supervised CAD systems hereafter) have two problems. The first problem is the difficulty of preparing training datasets. Considerable time and effort are required by even experts to correctly annotate numerous images with the information of diseases or lesions [9, 10]. The second is that the type of diseases that a supervised CAD system can correctly detect or diagnose is limited by the design of its training datasets. To develop a supervised CAD system that can detect various types of anomaly, it is necessary to prepare diverse types of anomalous data and to annotate the anomalies, which is also difficult [11, 12]. These problems can be addressed using a framework of unsupervised anomaly detection, that is, capturing the characteristics of normal images and detecting differences in the characteristics in the images assessed from those in the normal images. In this method, training requires only normal images and no lesion labels; furthermore, any type of abnormality can be detected.

Despite the above advantages, unsupervised anomaly detection is a technically challenging task and had not been widely applied to medical images. However, with the recent development of unsupervised methods in deep learning, several works on unsupervised anomaly detection in medical images have emerged [13–18]. These have employed autoencoders, especially variational autoencoders (VAEs) [19], or generative adversarial networks (GANs) [20], which are the most well-known classes of DNN-based unsupervised learning models. AnoGAN [13], an unsupervised anomaly detection framework based on a GAN, requires a time-consuming iterative process to calculate the inverse mapping of the generator for anomaly detection. VAE-based methods do not have this problem, but in general, VAEs generate more blurry images than GANs [21, 22]. In some recent papers, models combining an autoencoder and a GAN to utilize their advantages have been presented [22–24]. Baur C et al. [14] also reported an unsupervised anomaly detection/segmentation method based on a VAE–GAN model, targeting multiple sclerosis lesions in brain MR images. Very recently, Tang et al. [17] proposed an unsupervised anomaly detection method for chest radiographs using a hybrid model of a traditional (not variational) autoencoder and a GAN.

In this paper, we present an unsupervised anomaly detection method based on VAE-GAN and demonstrate its ability to detect various lesions using a large chest radiograph dataset. The contributions of this research are as follows:

• Unlike the supervised methods widely used in CAD for chest radiographs, our VAE-GAN-based unsupervised method can detect any kind of lesions and does not require any abnormal images and lesion labels for training.
• We achieved both anomaly detection based on Gaussian latent vectors derived from VAE and fine visualization of anomalies derived from GAN.

## Materials and Methods

### Overview

Here, we describe an overview of our anomaly detection method using a VAE-GAN. This anomaly detection is performed via the VAE part of the model, and the GAN part mainly contributes to improving image quality.

**Fig. 1:**
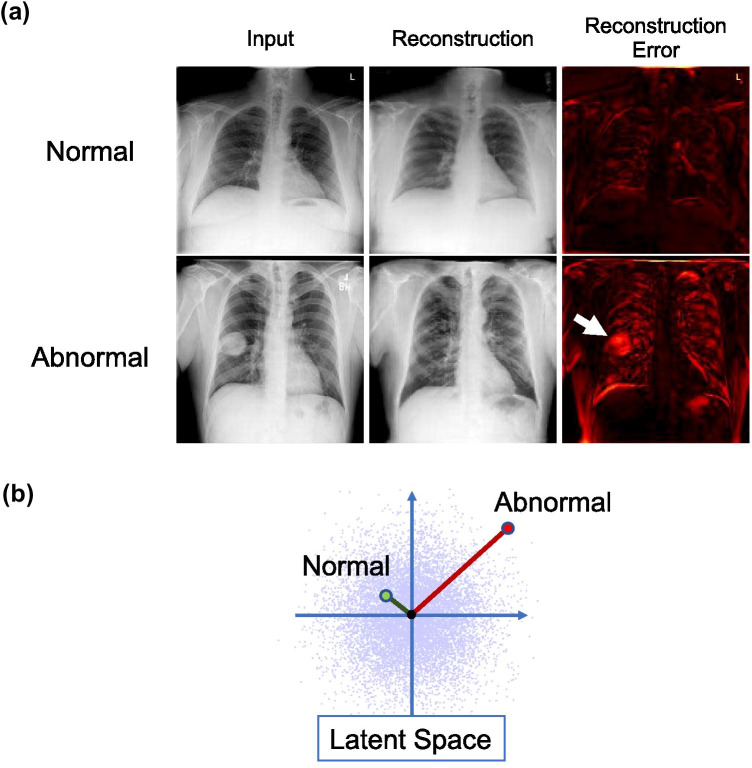
Overview of our anomaly detection system. (**a**) Anomaly detection based on reconstruction error. The anomaly (a lung mass in this figure) disappears after the reconstruction, and the total reconstruction error of an abnormal image is expected to be larger than that of a normal image. (**b**) Anomaly detection using code norm. Abnormal images will be out of the distribution of the normal images in the latent space (the standard Gaussian distribution ideally) and farther from the origin than normal ones. The 128-dimensional latent space is drawn as two-dimensional for the explanation

A VAE is a network that maps an input image to a low-dimensional vector called a latent code and then generates (or “reconstructs”) an output image from it. A VAE is trained so that the reconstructed image is as close as possible to the input image. In our method, the VAE is trained using a dataset consisting only of normal chest radiographs. This VAE will then be able to correctly reconstruct a normal chest radiograph. However, when it tries to reconstruct a radiograph with some anomaly, its output will be a somewhat "normal-like" reconstruction and the anomaly will disappear (Fig. 1a). Therefore, anomaly detection can be performed by taking the difference between the input image and the reconstructed image (hereinafter referred to as the reconstructed error).

In addition, the latent codes described above are trained to follow a standard normal distribution. Therefore, anomaly detection can be performed by regarding the latent codes close to the origin as normal and those far from the origin as abnormal (Fig. 1b).

### Dataset

We used a publicly available chest radiograph dataset: the Radiological Society of North America (RSNA) Pneumonia Detection Challenge dataset [25] (hereafter, the RSNA dataset). This dataset comprises 30,000 frontal view chest radiographs, with each image labeled as “Normal,” “No Opacity/Not Normal,” or “Opacity” by one to three board-certified radiologists. The Opacity group consists of images with opacities suspicious for pneumonia, and the No Opacity/Not Normal group consists of images with abnormalities other than pneumonia. The details of the RSNA dataset are shown in Table 1. The total number of images was smaller than 30,000 because the RSNA dataset includes some invalid images such as abdominal or lateral chest images; thus, they were excluded from the labeling [25]. All images were resized into 256 × 256 from the original size of 1024 × 1024 by Lanczos resampling.

**Table 1:** Details of the RSNA dataset

		Normal	Lung opacity	No lung opacity/not normal	Total
Age (Year)	Range	2–91	1–92	1–92	1–92
	Mean (SD)	45.0 (16.3)	49.4 (16.4)	45.6 (17.5)	47.0 (16.8)
Gender	Male	5496	4158	7226	16,880
	Female	4294	2948	5562	12,804
View position	PA	7995	1614	6520	16,129
	AP	1795	5492	6268	13,555
Total		9790	7106	12,788	29,684

*SD* standard deviation, *PA* posteroanterior, *AP* anteroposterior

We split this RSNA dataset into three subsets: the training, validation, and test datasets. The training dataset was used to train our model. The main feature of this study is that the training dataset consists only of normal images. The test dataset was used for the final performance evaluation. The validation dataset is a performance evaluation dataset separate from the test set and was used to determine the optimal number of training epochs. These two datasets contain both normal and abnormal images. We randomly sampled 70% (6,853/9,790) of the Normal images as the training dataset and randomly split the remaining images into the validation and test datasets in a ratio of 1:2 (7610 and 15,221). This random subsampling was performed ten times for cross-validation, which is described later. The details of this random splitting are shown in Table 2.

**Table 2:** Details of splitting dataset in our study

	Training	Validation	Test	Total
Normal	6853	979	1,958	9,790
Abnormal*	0	6631	13,263	19,894
Total	6853	7610	15,221	29,684

*“No lung opacity/not normal” or “lung opacity”

The RSNA dataset is a subset of the National Institutes of Health (NIH) Chest X-Ray dataset [26], which contains 112,120 frontal chest radiographs. The original NIH dataset also includes per-image labels of 14 thoracic diseases; however, these are far less accurate than the RSNA dataset because they were not annotated by human experts but automatically generated from radiological reports through natural language processing techniques. Thus, we used the RSNA dataset rather than the entire NIH dataset to ensure the accuracy of evaluation.

### Formularization of Anomaly Detection

**Fig. 2:**
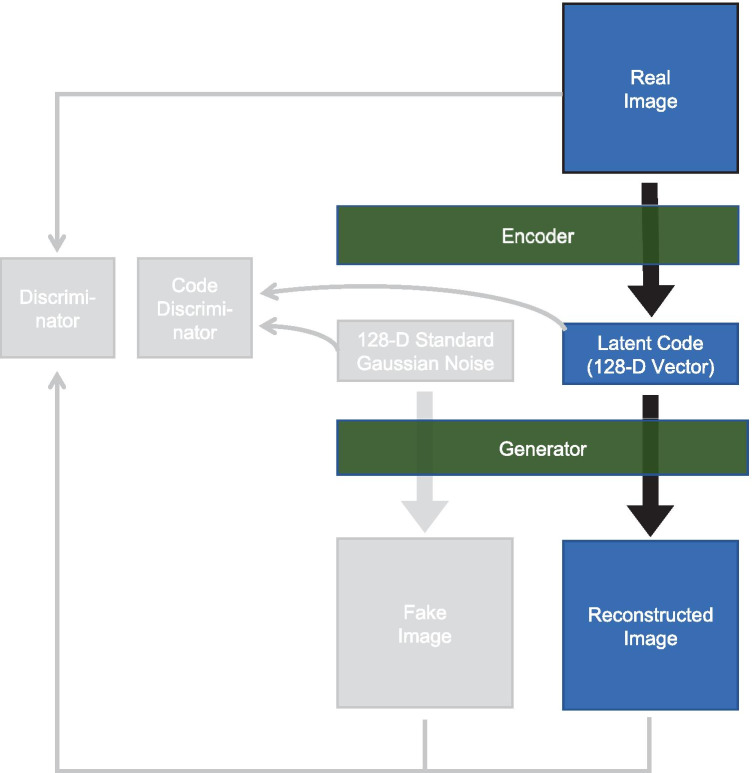
Illustration of α-GAN model. The grayed-out components are used only for training and are not used for our anomaly detection method. 128-D: 128-dimensional

We employed an auto-encoding GAN (α-GAN) [22] framework in our anomaly detection method. This is a combination of a GAN and a VAE and consists of four DNNs, an encoder, a generator, a discriminator, and a code discriminator (Fig. 2). The architectures of the networks are shown in Table 3. The encoder encodes an input image into a latent code, which is a 128-dimensional vector in our model, and the generator generates an image from a latent code. The encoder and the generator compose an autoencoder, which can reconstruct its own input image. These networks are trained so as to minimize the difference between input images and their reconstructions. The discriminator tries to discriminate generated images from real images in order to encourage the generator to generate images indistinguishable from the real images. The code discriminator similarly makes the distribution of latent codes closer to the standard Gaussian distribution. See Rosca et al. [22] for more details.

**Table 3:** Architectures of the networks

Generator				Encoder		
Layer	Activation	Output shape		Layer	Activation	Output shape
(Latent vector)		128		(Input Image)		256 × 256 × 1
Linear	LReLU	4 × 4 × 512		Convolution 1 × 1	LReLU	256 × 256 × 8
Upsampling		8 × 8 × 512		Convolution 3 × 3	LReLU	256 × 256 × 16
Convolution 3 × 3	LReLU	8 × 8 × 256		Downsampling		128 × 128 × 16
Upsampling		16 × 16 × 256		Convolution 3 × 3	LReLU	128 × 128 × 32
Convolution 3 × 3	LReLU	16 × 16 × 128		Downsampling		64 × 64 × 32
Upsampling		32 × 32 × 128		Convolution 3 × 3	LReLU	64 × 64 × 64
Convolution 3 × 3	LReLU	32 × 32 × 64		Downsampling		32 × 32 × 64
Upsampling		64 × 64 × 64		Convolution 3 × 3	LReLU	32 × 32 × 128
Convolution 3 × 3	LReLU	64 × 64 × 32		Downsampling		16 × 16 × 128
Upsampling		128 × 128 × 32		Convolution 3 × 3	LReLU	16 × 16 × 256
Convolution 3 × 3	LReLU	128 × 128 × 16		Downsampling		8 × 8 × 256
Upsampling		256 × 256 × 16		Convolution 3 × 3	LReLU	8 × 8 × 512
Convolution 3 × 3	LReLU	256 × 256 × 8		Downsampling		4 × 4 × 512
Convolution 1 × 1	Tanh	256 × 256 × 1		Linear		128
Discriminator				Code Discriminator		
Layer	Activation	output shape		Layer	Activation	output shape
(Input Image)		256 × 256 × 1		(Latent vector)		128
Convolution 1 × 1	LReLU	256 × 256 × 8		Linear	LReLU	1500
Convolution 3 × 3	LReLU	256 × 256 × 16		Linear		1
Downsampling		128 × 128 × 16				
Convolution 3 × 3	LReLU	128 × 128 × 32				
Downsampling		64 × 64 × 32				
Convolution 3 × 3	LReLU	64 × 64 × 64				
Downsampling		32 × 32 × 64				
Convolution 3 × 3	LReLU	32 × 32 × 128				
Downsampling		16 × 16 × 128				
Convolution 3 × 3	LReLU	16 × 16 × 256				
Downsampling		8 × 8 × 256				
Convolution 3 × 3	LReLU	8 × 8 × 512				
Downsampling		4 × 4 × 512				
Linear		1				

*LReLU* leaky rectified linear unit, *Tanh* hyperbolic tangent

First, we trained these networks with the training dataset, consisting of only normal chest radiographs. As mentioned in the subsection “Overview,” we can measure the anomaly score of an input image **x** by calculating the sum of the differences between the pixel values of the original and reconstructed images (Fig. 1a):

1 $$\begin{array}{c}reconstruction\_error({\mathbf{x}})= {\Vert {\mathbf{x}}-\mathrm{Gen}\left(\mathrm{Enc}\left({\mathbf{x}}\right)\right)\Vert }_{1}\end{array}$$

where $\mathrm{Gen}$ and $\mathrm{Enc}$ are the generator and the encoder respectively and ${\Vert \bullet \Vert }_{1}$ is the pixelwise L1 norm. This method yields not only a per-image anomaly score but a per-pixel anomaly score, which is useful for visualizing anomalies.

We can also measure the per-image anomaly score by simply calculating the Euclidean norm of the latent code.

2 $$\begin{array}{c}code\_norm({\mathbf{x}})=\Vert \mathrm{Enc}\left({\mathbf{x}}\right)\Vert \end{array}$$

Since outputs of the encoder for the normal chest radiographs ideally follow a multivariate standard Gaussian distribution, we can measure the anomaly degree of the input image by **x** calculating the distance between the corresponding latent vector $\mathrm{Enc}\left({\mathbf{x}}\right)$ and the origin (Fig. 1b).

### Model Implementation and Training Details

We implemented our model using Chainer (https://chainer.org/) version 4.4.0 as a deep neural network framework. We used a supercomputer system (Reedbush-H) in our institution, which consists of 120 computing nodes equipped with two GPUs (Tesla P100, NVIDIA Corporation, Santa Clara, CA). The batch size was set to 10. We used the Adam optimizer with *α* = 0.0005, *β*_1_ = 0.5, and *β*_2_ = 0.9, similar to in the α-GAN paper [22]. We employed a progressive growing technique [27] to stabilize the training of the generator, the encoder, and the discriminator. The training procedure was as follows:

- 1. We first started with an image size of 4 × 4 and trained only the linear layers of these networks until the first epoch ended.
- 2. Then we upsized the resolution to 8 × 8 and faded in the next (upsampling/downsampling and convolutional) layers gradually until the second epoch ended and continued training in order to stabilize them until the third epoch ended.
- 3. We similarly added the layers progressively until the resolution became 256 × 256.

### Evaluation

As a visual assessment of our per-pixel and per-image anomaly detection method, we show some examples of anomaly location visualization by the reconstruction error method and images of the highest and the lowest code norm scores. For quantitative evaluation, we performed receiver operating characteristic (ROC) analysis of the image-level anomaly detection performance of the reconstruction error and the code norm anomaly scores. The images labeled as Normal in the RSNA dataset were regarded as negative and the rest as positive. To evaluate the performance difference depending on the class of anomalies, we also performed ROC analysis with positive samples limited to each class (Lung Opacity or No Lung Opacity/Not Normal). The training and this quantitative evaluation were repeated ten times for each random split of the dataset as Monte Carlo cross-validation, and the area under the ROC curve (AUROC) values are reported with 95% confidence intervals (CI). The optimal number of training epochs was determined using the validation set. First, the training session was run for 50 epochs. At the end of each epoch, the model was saved, and the AUROC values of the code norm scores were calculated for the validation set. Then, the model with the best validation scores was finally used for evaluation.

## Results

### Visual Assessments

**Fig. 3:**
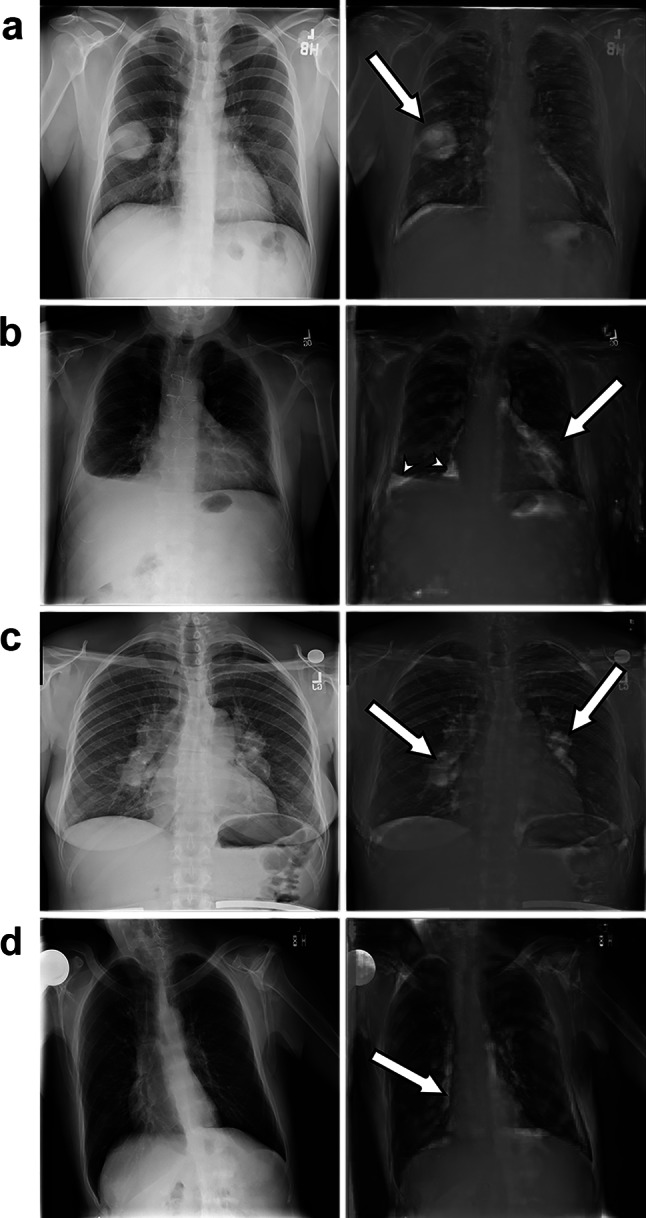
Examples of anomaly location visualization. The original images are shown on the left side and the reconstruction error images overlaid on the original images are shown on the right side. **a** Mass. **b** Cardiomegaly (arrow) and pleural effusion (arrowheads). **c** Bilateral hilar lymphadenopathy. **d** Dextrocardia

Figure 3 shows examples of anomaly location visualization using the reconstruction error. It can be seen that our system could correctly localize various lesions or anomalies, namely, a lung mass, cardiomegaly, pleural effusion, bilateral hilar lymphadenopathy, and even dextrocardia. More examples are available in Supplemental Materials.

**Fig. 4:**
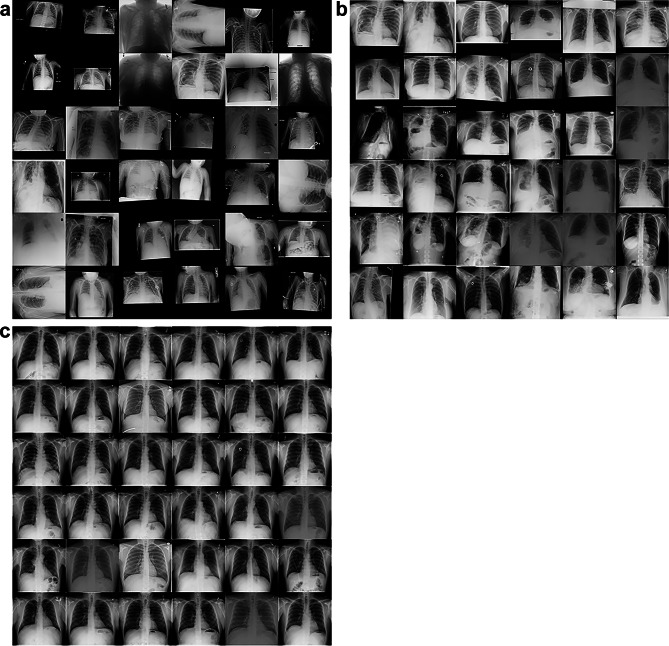
Images with the **a**, **b** highest and **c** lowest code norm anomaly scores. The images in **b** are limited to the posteroanterior adult chest images and incorrectly rotated or color-inverted images are also excluded

Figure 4 shows radiographs with the highest and lowest code norm scores in the test dataset. The highest-scored images (Fig. 4a) include inappropriate chest radiographs such as incorrectly rotated or color-inverted ones and images with small and/or off-centered fields of view, mostly in those from children. Figure 4b shows the highest-scored posteroanterior adult chest radiographs, excluding incorrectly rotated or color-inverted radiographs. Most of these images have various bulky lesions or anomalies such as a large mass, pneumonia involving the entire lung, a large amount of pleural effusion, and thoracic deformation probably due to thoracoplasty. By contrast, the lowest-scored images shown in Fig. 4c are all similar. Most of them have no bulky lesions, have the normal form of thoraces and are correctly positioned.

### Quantitative Performance of Anomaly Detection

**Fig. 5:**
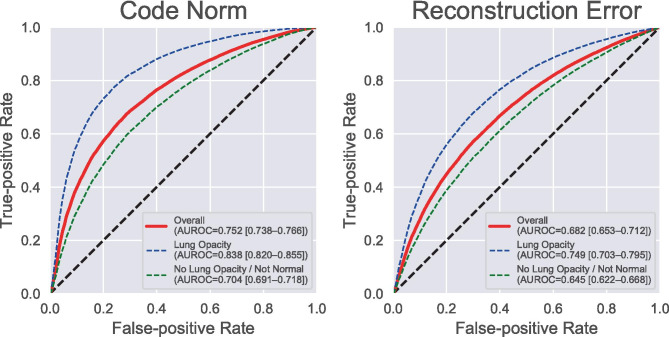
Receiver operating characteristic (ROC) curves for per-image anomaly detection tasks. Each value in parentheses represents the area under the corresponding ROC curve and its 95% confidence interval. AUROC area under the ROC curve

The average ROC curves of the per-image anomaly detection task are shown in Fig. 5 with the AUROC values and their 95% CIs. The anomaly detection method with the code norm score on average detected 67.2% of the abnormal chest radiographs with a false-positive rate of 28.5%. The AUROC was 0.752 (95% CI, 0.738–0.766). The AUROCs for each abnormal label (Opacity and No Opacity/Not Normal) were 0.838 (0.820–0.855) and 0.704 (0.691–0.718), respectively. The reconstruction error method showed worse performance than the code norm method, with an overall AUROC of 0.630 (0.579–0.682). Each training session took an average of 10,768 s, and each evaluation session took an average of 183 s (12 ms/image), with a Tesla P100 GPU (NVIDIA Corporation, Santa Clara, CA).

## Discussion

We have shown that our unsupervised anomaly detection method can successfully detect and localize lesions in chest radiographs. In contrast to supervised CAD systems requiring images and annotations of target diseases or lesions for training, our system requires only normal chest radiographs and no annotations, making it easy to create a training dataset. In addition, this method can also detect various lesions or anomalies, in contrast to supervised CAD systems, which can generally detect only specific lesions. In addition to pathological anomalies, our method can even detect technical anomalies such as inappropriate rotation, inversion, and positioning, as shown in Fig. 4a. This means our method may also be applied to detect technical errors in image acquisition, as well as for diagnostic assistance. Moreover, because this method does not require any specific processing for the targets, it can be easily applied to any target, not only to chest radiographs but to any organ and even any modality. We can develop a CAD for any target by simply gathering "normal" images.

In clinical practice, it is often the case that unexpected diseases or lesions are found in patients. Whereas a disease-specific supervised CAD system can hardly detect such unexpected disease, our method can easily detect them by finding "not normal" features. This process of learning the features of normal images and detecting the difference from them is similar to what radiologists do when assessing radiological images. Training for radiologists starts with studying the normal anatomy and familiarizing them with the features of normal images. When assessing an image, radiologists first look for abnormal findings and then determine what they are. Human radiologists cannot make a diagnosis unless they find an abnormality. Our system will prevent us from oversights and help in the first step of diagnosis.

Very recently, Tang et al. [17] also proposed an unsupervised anomaly detection method for chest radiographs using a hybrid model of an autoencoder and a GAN, and reported an AUROC of 0.805, although this value cannot be directly compared with our results because of the difference in the datasets. A major difference between our method and that of Tang et al. is that we use a VAE, while Tang et al. used a traditional, not variational, autoencoder. The benefit of using a VAE over a traditional autoencoder is that we can make the latent variables follow the standard Gaussian distribution, which enables simple latent–variable-based anomaly detection (called the “code norm” method in our paper). A traditional autoencoder does not assume any distribution over the latent variables; thus, it is difficult to perform anomaly detection based on the latent variables as it is. For the model by Tang et al. it is necessary to train an additional encoder, which encodes fake images to latent variables, to utilize latent variables for anomaly detection. Our method has also succeeded in generating larger and higher-quality reconstruction images than that of Tang et al., which provides fine anomaly visualizations (see Figs. 1 and 3).

We found that the code norm score performs better than the reconstruction error score in the per-image anomaly detection task in our experiments. We observed that the reconstructed images have a slight deviation from the original images, especially in the thorax and body contour (see Figs. 1a and 3), which may degrade the reconstruction error score. To address a similar problem in the reconstruction of brain MR images, Baur et al. [14] performed various postprocessing methods such as the use of a median filter, erosion, and removal of small connected components. The code norm method is free from this misregistration problem and does not require such complicated postprocessing, but as it is, it has the disadvantage that it is difficult to obtain a visual explanation for anomaly detection. Applying recent visual explanation techniques for DNNs such as Grad-CAM [28] and SmoothGrad [29] to the code norm method may help identify abnormal sites more accurately, which will be our future work.

This method has a limitation in that it provides discrimination only between normal and abnormal images; it can detect any anomaly but cannot diagnose it. It detects any features in the assessed images that are different from those in training images, regardless of what they are and whether they are clinically significant or not. Thus, this approach does not replace the human doctor, but is rather a tool to help detect lesions and prevent oversights. Another limitation is its performance in anomaly detection. Unsupervised anomaly detection techniques often perform worse than supervised techniques [12] in the detection of specific objects. For example, CheXNet [1], one of the state-of-the-art supervised CAD systems for chest radiographs, has achieved an AUROC of greater than 0.9 for some diseases. This is better than our AUROCs of 0.7–0.8, although these values cannot be compared directly because of the difference in the tasks and datasets used. Further development of unsupervised anomaly detection techniques and/or a combination with supervised techniques will improve in the performance of anomaly detection. Our study also lacks a sufficient quantitative performance evaluation for various diseases or anomalies. The RSNA dataset does not have detailed labeling for findings other than lung opacity; therefore, we cannot perform per-disease ROC analysis for them at this time. We hope to prepare an evaluation dataset and perform further analysis in the future.

## Conclusion

We have proposed an unsupervised anomaly detection system based on a VAE–GAN model and shown that it can successfully detect various diseases or anomalies in chest radiographs by training only with the normal images. Although unsupervised anomaly detection is still a challenging task, it has a wide range of potential applications that may spread to various fields with the development of unsupervised deep learning techniques. Our future work will focus on the improvement of performance in anomaly detection and visualization, in which we aim to clinically apply an all-purpose initial screening tool for any type of anomaly and even for any modality including 3D images.

## Supplementary Information

Below is the link to the electronic supplementary material.

Supplementary file1 (PDF 29421 KB)
