# Supplementary material for: Unsupervised Deep Anomaly Detection in Chest Radiographs
Source: J Digit Imaging. 2021 Feb 8;34(2):418–27. doi: 10.1007/s10278-020-00413-2 (PMC8289984; doi:10.1007/s10278-020-00413-2)
Supplement: Supplementary file 1 — Supplementary file1 (PDF 29421 KB) [file 10278_2020_413_MOESM1_ESM.pdf]

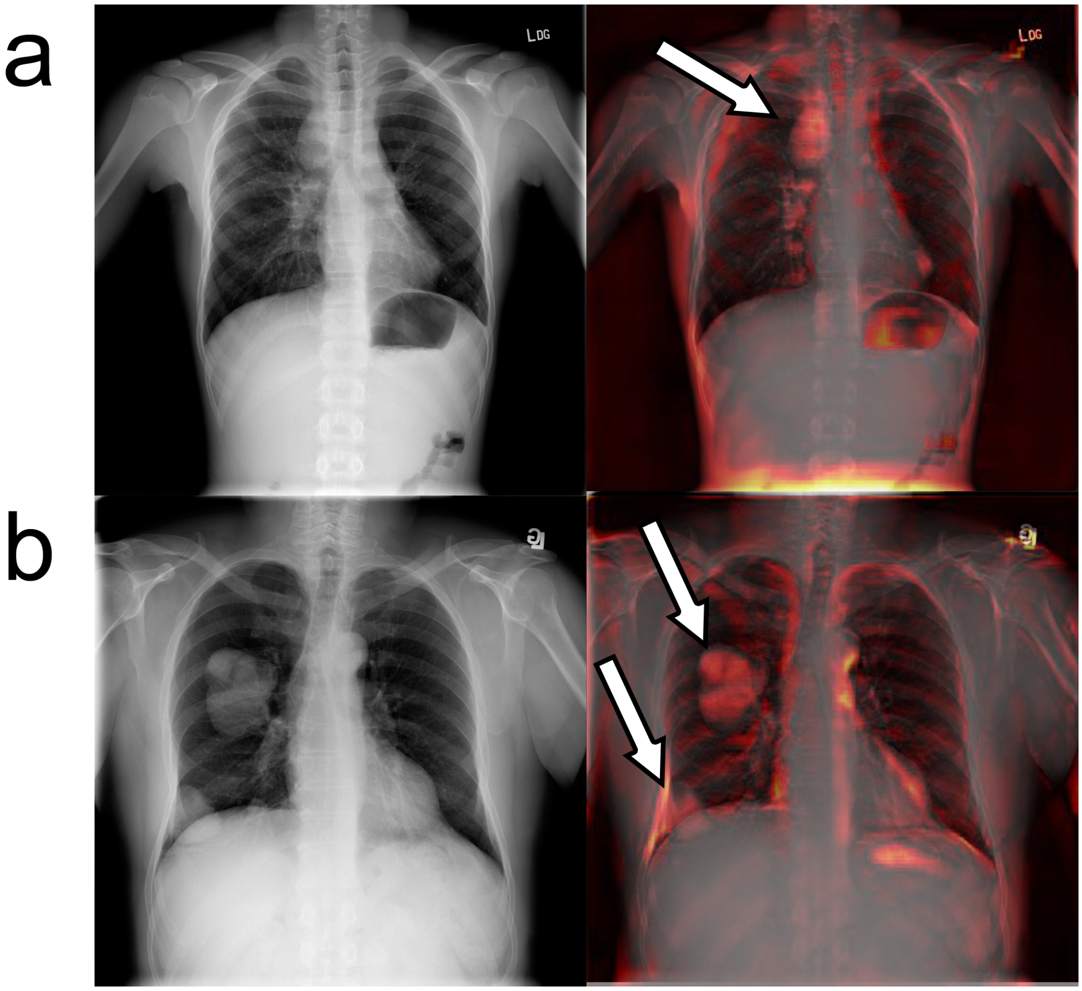

Lung masses (arrows).

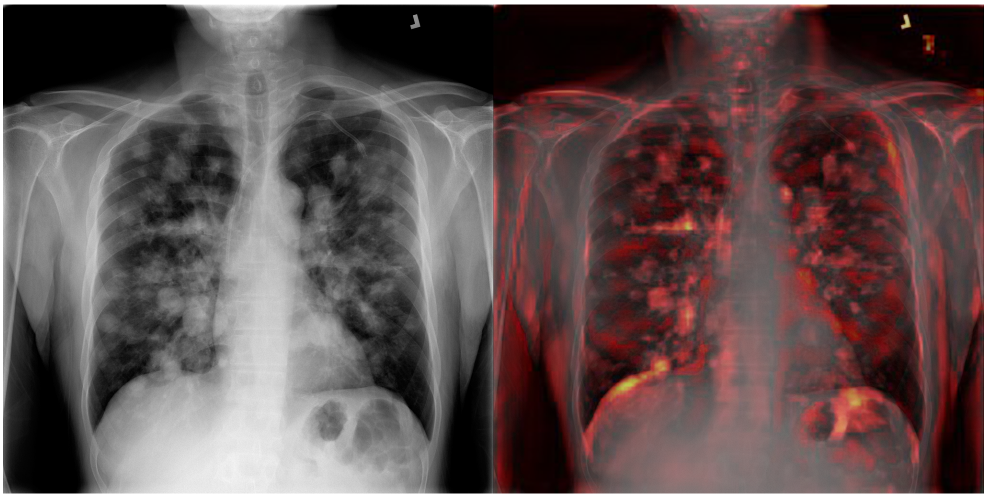

Multiple nodules.

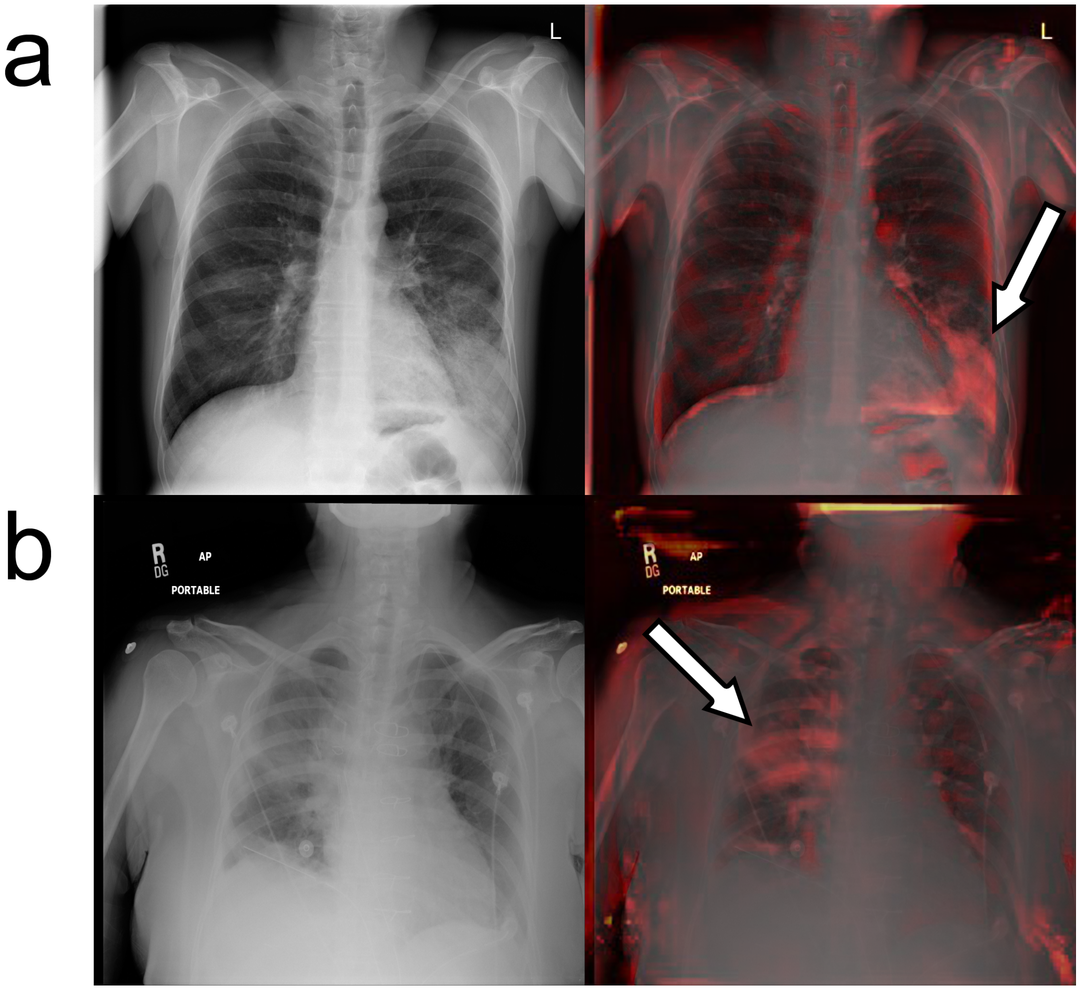

Lung opacities (arrows).

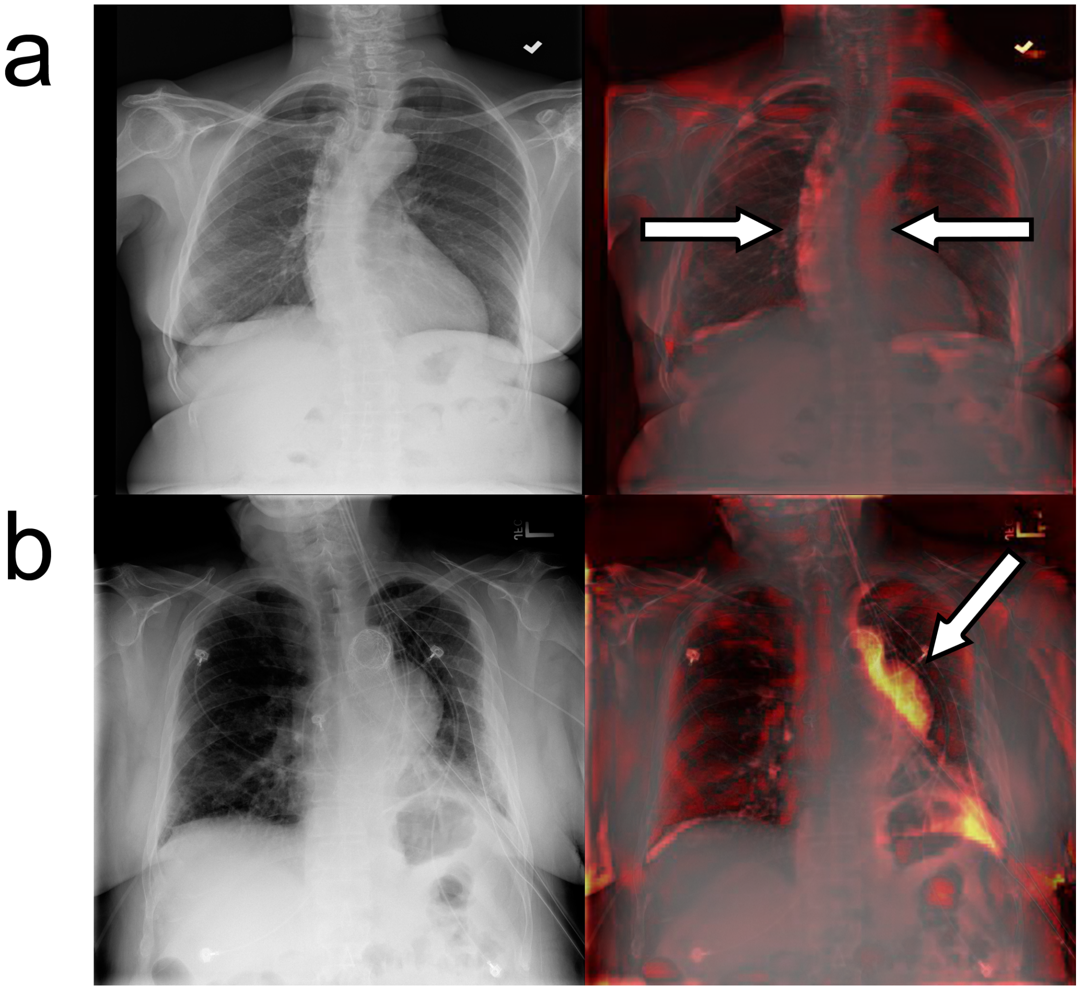

(a) Scoliosis (arrows) and (b) Thoracic aortic aneurysm (arrow).

a

b

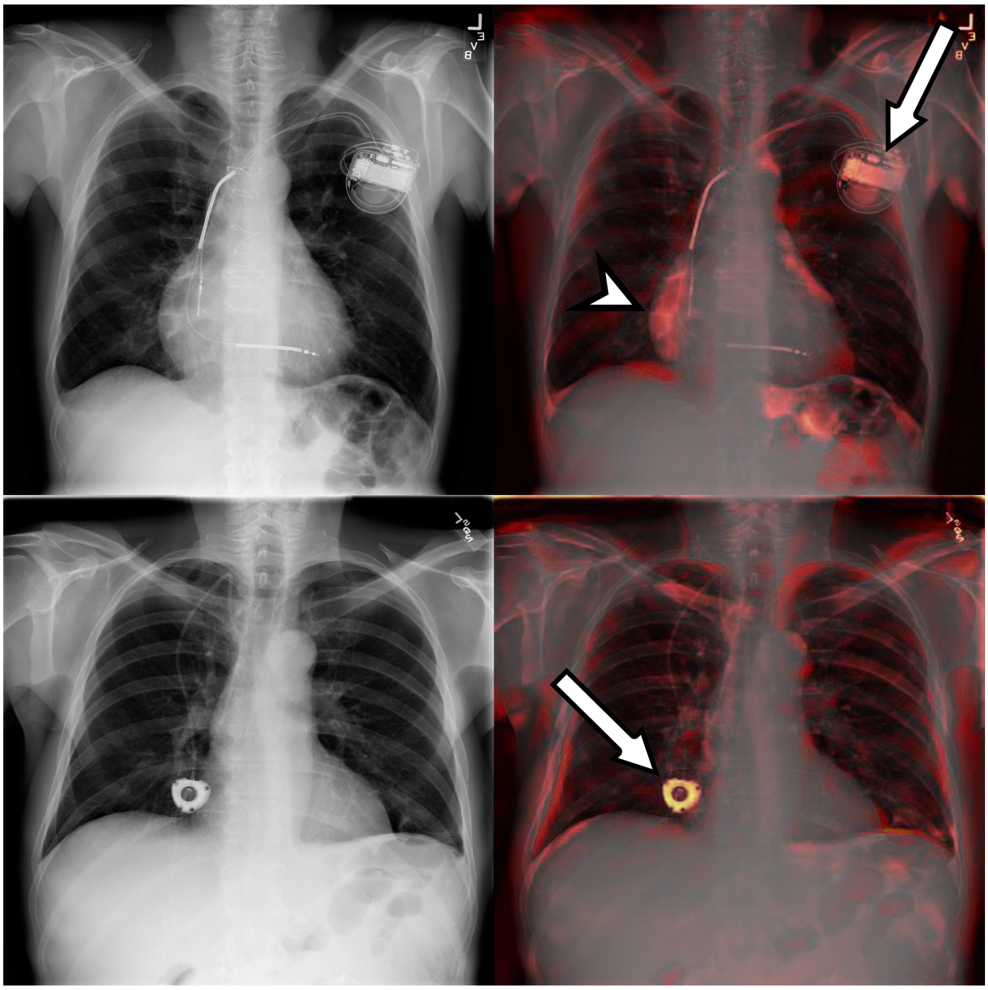

Medical implants.

(a) Cardiac pacemaker (arrow) and cardiomegaly (arrowhead). (b) chest port (arrow).
